# Hepatocyte Growth Factor (HGF) Promotes Peripheral Nerve Regeneration by Activating Repair Schwann Cells

**DOI:** 10.1038/s41598-018-26704-x

**Published:** 2018-05-29

**Authors:** Kyeong Ryang Ko, Junghun Lee, Deokho Lee, Boram Nho, Sunyoung Kim

**Affiliations:** 1 School of Biological Sciences, Seoul National University, Seoul, 08826 Korea; 2 Viro Med, Co., Ltd, Seoul, 08826 Korea

## Abstract

During the peripheral nerve regeneration process, a variety of neurotrophic factors play roles in nerve repair by acting on neuronal or non-neuronal cells. In this report, we investigated the role(s) of hepatocyte growth factor (HGF) and its receptor, c-met, in peripheral nerve regeneration. When mice were subjected to sciatic nerve injury, the HGF protein level was highly increased at the injured and distal sites. The level of both total and phosphorylated c-met was also highly upregulated, but almost exclusively in Schwann cells (SCs) distal from the injury site. When mice were treated with a c-met inhibitor, PHA-665752, myelin thickness and axon regrowth were decreased indicating that re-myelination was hindered. HGF promoted the migration and proliferation of cultured SCs, and also induced the expression of various genes such as GDNF and LIF, presumably by activating ERK pathways. Furthermore, exogenous supply of HGF around the injury site, by intramuscular injection of a plasmid DNA expressing human HGF, enhanced the myelin thickness and axon diameter in injured nerves. Taken together, our results indicate that HGF and c-met play important roles in Schwann cell-mediated nerve repair, and also that HGF gene transfer may provide a useful tool for treating peripheral neuropathy.

## Introduction

When the peripheral nerve gets wounded, SCs formed myelin structure are dedifferentiated and initiate the regeneration process^1^. Dedifferentiated SCs begin to produce not only cytokines and chemokines such as TNF-α, LIF, and CCL2/MCP-1, but also secret various neurotrophic factors, for example, GDNF, NGF, and VEGF to promote axon elongation and activate other cells for nerve repair^1–3^. In addition, SCs directly interact with the peripheral nerves to guide regenerating axons to the distal nerve. Once axons regrow, SCs in close proximity to newly regenerated neurons bind to axons and start the re-myelination process. As a result, axons regrow and innervate to their target tissues, and new myelin structures are formed^4,5^.

A variety of neurotrophic factors have been studied for their involvement in nerve regeneration. The most extensively studied genes include GDNF, NGF and VEGF for their functions and actions in the peripheral nerve regeneration process^6–9^. These growth factors are highly expressed in injured nerve and activate neuronal and non-neuronal cells for repair of the injured nerves^10^. For instance, VEGF directly increases the neuron survival rate and axon outgrowth. VEGF also promotes the vascularization of the injured nerve to reconstitute its microenvironment, leading to the facilitation of the nerve repair process^11–13^.

Hepatocyte growth factor (HGF) is another angiogenic factor that has been reported to work on the nervous system directly or indirectly^14^. HGF has been shown to promote angiogenesis, cell survival, cell migration and anti-inflammation in a variety of cell types^15^, and also produce neurotrophic effects in both CNS and PNS^14^. This multifunctional protein is secreted mainly by mesenchymal lineage cells, and receptor tyrosine kinase c-met is the only known cellular receptor^15^. In the case of PNS, HGF can enhance survival and axon outgrowth of cultured motor neurons^16–19^, and it is also known to interact with NGF to exert neurotrophic effects on sensory neurons^20^. Recently, it has been reported that Gab2 protein is required for the migration and proliferation of SCs and HGF could induce the activation of Gab2 to show such cellular effects^21^. However, whether c-met receptor has any role in peripheral nerve injury is largely unknown.

In this study, we investigated the role(s) of HGF and c-met in peripheral nerve regeneration, using the mouse nerve crush model. It was found that HGF expression was highly induced at the nerve injury site, while the level of phosphorylated c-met as well as total c-met activation was greatly increased mainly in SCs distal from the injury site. When the c-met inhibitor (PHA665752) was administrated to mice, the re-myelination process was suppressed. Treatment of rat primary SCs with recombinant human HGF protein enhanced both migration and proliferation. HGF also increased the expression of neurotrophic factors and inflammatory cytokines such as GDNF, LIF, and TNF-α. We also found that HGF could activate downstream signaling pathways such as ERK/RSK, AKT and S6K, but not mTOR, STAT3 or JNK pathways. When HGF was exogenously introduced around the injury site by intramuscular injection of plasmid DNA engineered to express HGF, myelin thickness of injured nerve was significantly improved, indicating that the nerve regeneration process was facilitated. Taken together, our data suggested that HGF could induce the activation of Schwann cells to promote re-myelination of damaged nerves, and that exogenous addition of HGF could expedite the nerve regeneration process.

## Results

### HGF expression is increased in injured peripheral nerves

**Figure 1:**
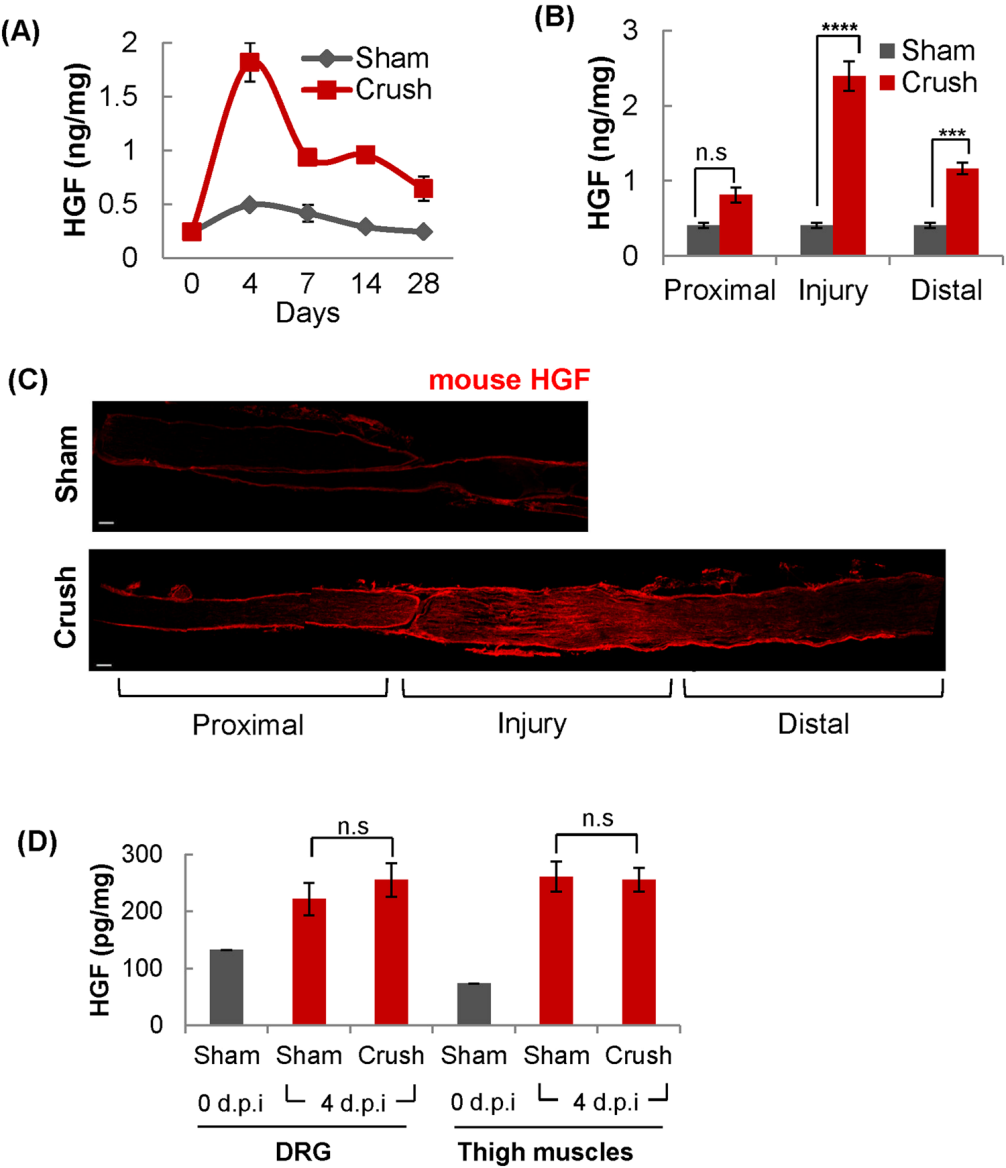
Increased expression of HGF in the nerve injury site. (**A**) Time kinetics of HGF expression in the sciatic nerve after nerve injury. Following nerve crush, total ipsilateral sciatic nerves were isolated at appropriate time points, and total proteins were analyzed by ELISA to measure the HGF protein level. (**B**) Spatial distribution of HGF expression in the injured sciatic nerve. The level of the HGF protein was measured in three different areas of the sciatic nerve (proximal, injury and distal sites) at 4 d.p.i by ELISA. ***p < 0.001, ****p < 0.0001, n.s = not significant (**C**) High level expression of HGF in the injured site of sciatic nerve. Ipsilateral nerves were isolated at 4 d.p.i and subjected to immunohistochemical assay using an antibody to HGF (red). Scale bar = 100 μm (**D**) HGF expression in DRG and thigh muscles detected at 4 d.p.i. The protein level of HGF was measured in thigh muscles at 0, and 4 d.p.i by ELISA. n = 3 for each group. n.s = not significant. Values represent the mean ± S.E.M.

To investigate the role(s) of HGF in peripheral nerve regeneration, we first analyzed the temporal and spatial pattern of HGF expression in the injured nerve, employing the mouse nerve crush model widely used in the study of peripheral nerve regeneration. After nerve crush was introduced, injured sciatic nerves were isolated at different time points, and the protein level of HGF was analyzed by ELISA. In a sham control, the sciatic nerve was exposed by muscle incision, but without nerve injury. As shown in Fig. 1A, the protein level of HGF was highly increased from the basal point (approximately ~0.24 ng/mg), reaching a peak (1.82 ng/mg of total sciatic nerve protein) at 4 days post injury (d.p.i). Thereafter, its expression level was decreased to 0.93 ng/mg at day 7. Even at day 28, however, the HGF level was higher by 2.6 fold than that of sham mice.

The spatial pattern of HGF expression after injury was also studied by measuring the level of HGF in three different areas of crushed nerves (proximal, injury and distal sites) by ELISA and immunohistochemistry (IHC) at 4 d.p.i. As shown in Fig. 1B, the level of the HGF protein was significantly increased in both the injury and distal sites of damaged sciatic nerves, with induction level always highest at the injury site. In the proximal area, there was little difference between sham and crushed mice. This spatial pattern of HGF expression was also confirmed by IHC assay in which the strongest positive HGF signal was detected at the injured site followed by the distal site (Fig. 1C).

Other tissues were also analyzed for the HGF protein. Total proteins were isolated at 0 and 4 d.p.i from the dorsal root ganglia (DRG) and thigh muscles around injured sciatic nerves followed by ELISA. At 4 d.p.i., the HGF level was slightly increased in both tissues compared to day 0, presumably due to the muscle incision procedure, but there was no difference between sham and crushed animals (Fig. 1D). Taken together, these data indicated that nerve injury induced HGF expression mainly at the injury and distal areas of the sciatic nerve, but not in the DRG or thigh muscles.

### High levels of phosphorylated c-met are produced mainly in the distal site of injured nerves

**Figure 2:**
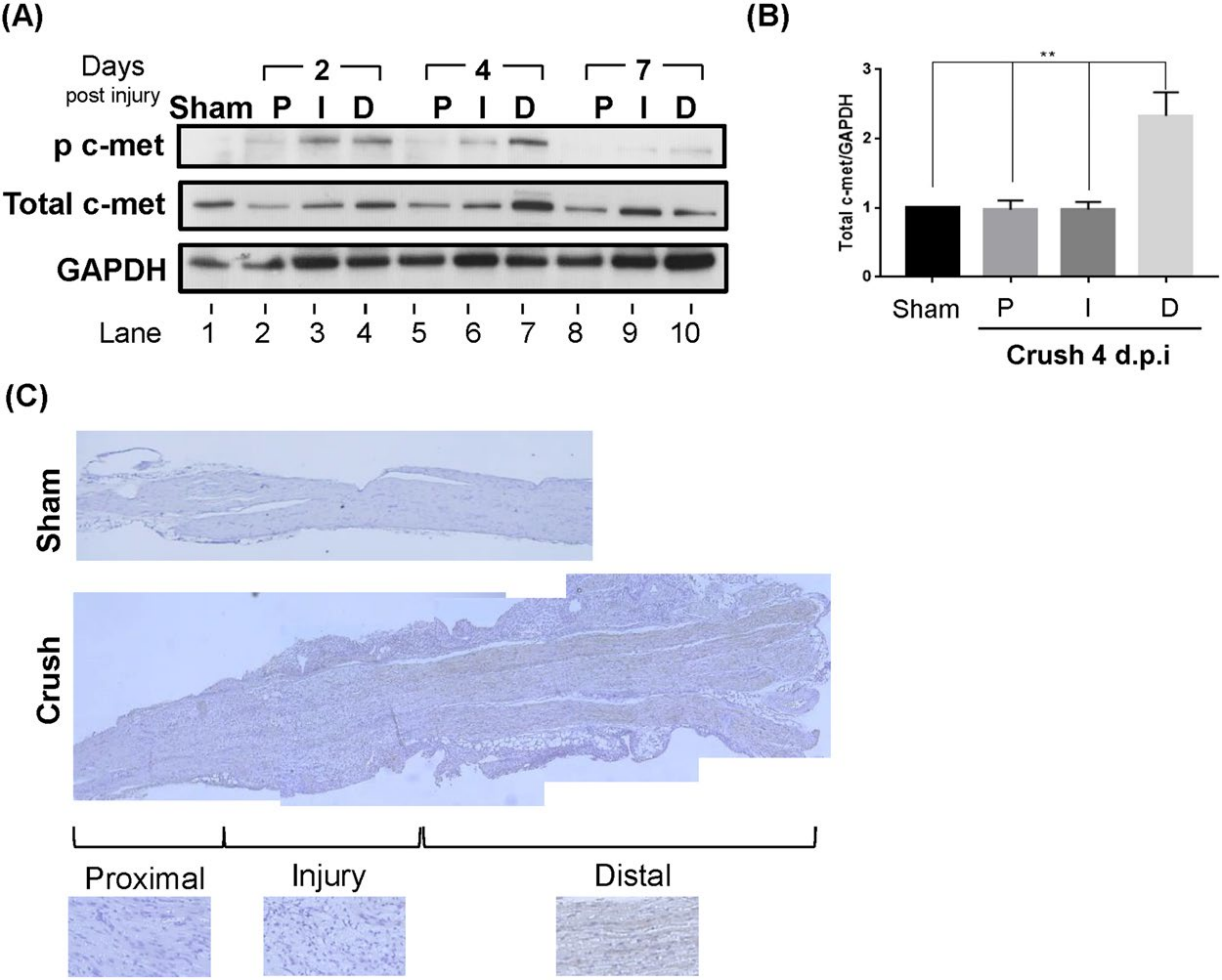
Expression and activation of c-met. (**A**) c-met expression in different areas of the injured nerve. After nerve crush, proximal (P), injury (I), and distal (D) regions were isolated at different time points followed by Western blot using an antibody to phosphorylated or total c-met. GAPDH was used as a loading control. Full-length blots are presented in Supplementary Fig. S4 (**B**) The bar graph shows the expression level of total c-met expression at crush 4 d.p.i. Full-length blots from three independent experiments are presented in Supplementary Figs S4A,B and S5. **P < 0.01. Values represent the mean ± S.E.M. (**C**) Immunohistochemical analysis of c-met receptor. Injured sciatic nerves were isolated at 4 d.p.i.

Since c-met is the only known receptor of HGF^15^, the expression pattern of c-met around injured nerves was also analyzed. The three sites of the sciatic nerve (proximal, injury, and distal) were isolated at different time points followed by Western blot analysis, using antibodies to phosphorylated or total c-met. The change in the level of phosphorylated c-met was evident. In the distal region, its level was increased from day 2, reached a peak at day 4, and became barely detectable at day 7 (Fig. 2A, compare lanes 1, 4, 7, and 10). In the injury site, the kinetics were a bit faster, with the peak level observed at day 2. In the proximal area, the level of phosphorylated c-met remained very low. The change in the level of total c-met was similar. In the distal region, it was increased from 2 d.p.i. and reached the maximum level at day 4 followed by a decrease at day 7 (Fig. 2A, compare lanes 1, 4, 7, and 10). No change was seen in the proximal area at all three time points.

The pattern of c-met expression at 4 d.p.i. was also analyzed by IHC; a high level of total c-met was clearly visible, but the signal intensity was lower at the injury site and virtually undetectable at the proximal region (Fig. 2C). Taken together, these data suggested that HGF produced from the nerve injury site might lead to the activation of c-met receptor present in distal region cells.

### Distal SCs express high levels of both total and phosphorylated c-met after nerve injury

**Figure 3:**
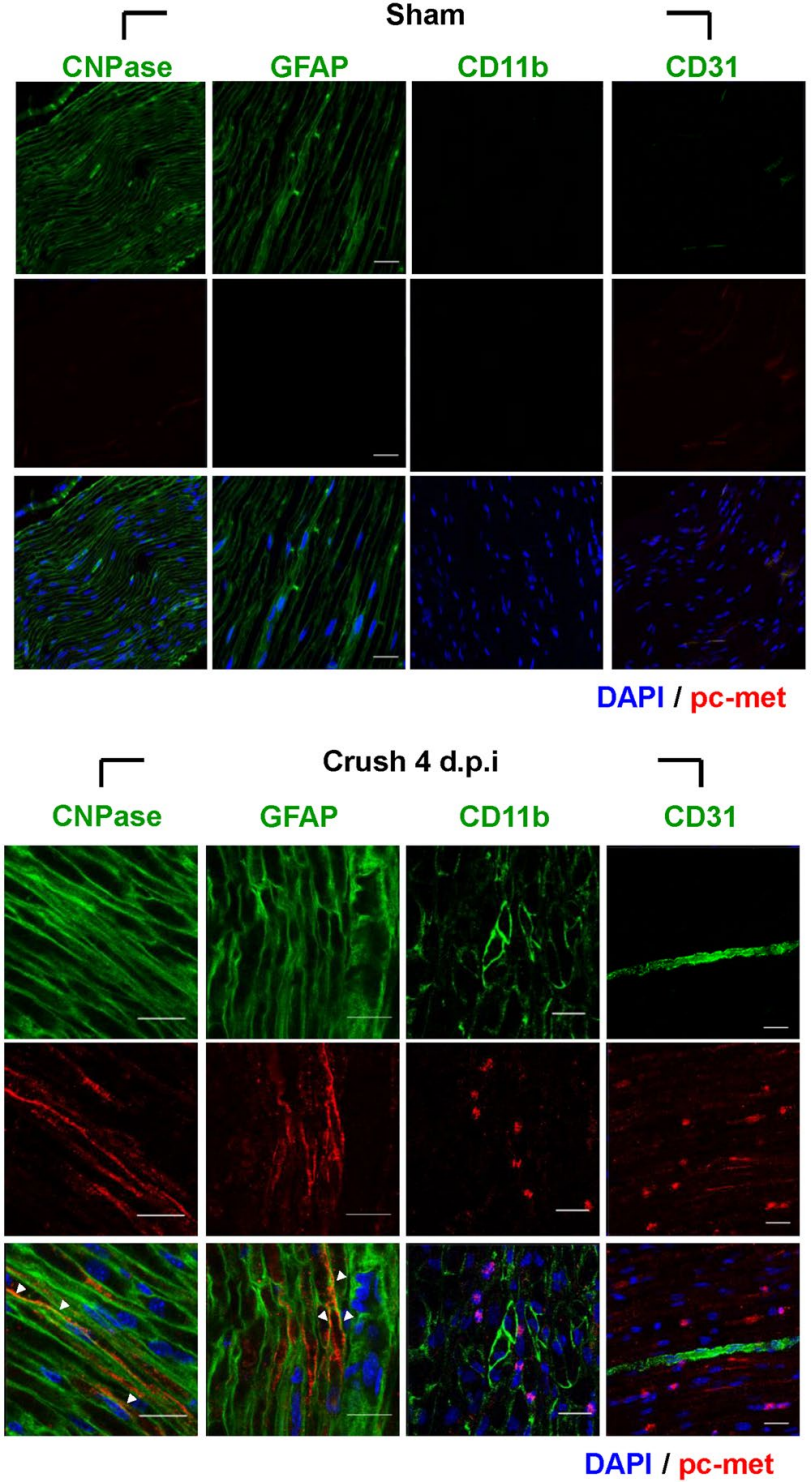
Identification of cell types expressing c-met. The distal sites of injured sciatic nerves were analyzed for various cell markers by immunohistochemistry assay using antibodies to CNPase and GFAP for SCs, CD11b for macrophages, CD31 for endothelial cells (all green), and phosphorylated c-met (red). The phosphorylated c-met was mainly merged with SCs marker (white arrow). Injured sciatic nerves were prepared at crush 4 d.p.i. Nuclei were counterstained with Hoechst (blue). n = 3 for each group. Scale bar = 20 μm.

A variety of different cell types are known to express c-met receptor and respond to HGF, including fibroblasts, endothelial cells, skeletal muscles, and neurons^22^. To identify the c-met expressing cell type(s) under the nerve injury situation, IHC was performed for cells around the distal sciatic nerve area by using antibodies to phosphorylated c-met, CNPase and GFAP for SCs, CD11b for macrophages, and CD31 for endothelial cells, respectively. As shown in Fig. 3, both activated and total c-met was detected mainly in distal SCs (Fig. 3, white arrow for activated c-met and Supplementary Fig. S1 for total protein). Endothelial cells also contained some positive signals for phosphorylated c-met, but they formed a small fraction among c-met positive cells. The c-met signal was not observed in macrophages. These data suggested that dedifferentiated SCs in the distal area were probably the major target of HGF produced from injured nerves.

### Axonal regeneration process is suppressed by c-met inhibitor

**Figure 4:**
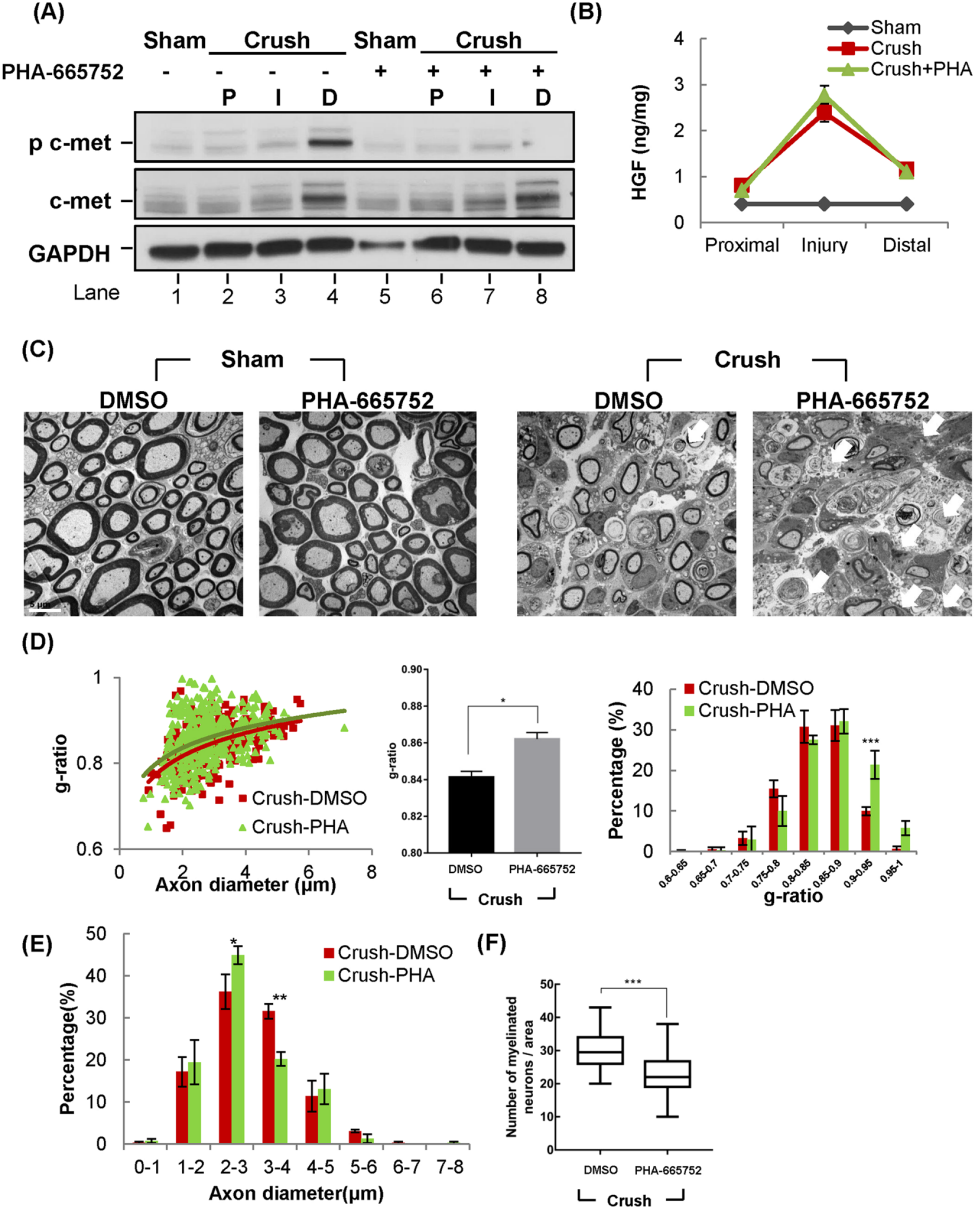
Nerve regeneration was inhibited by c-met inhibitor, PHA-665752. After sciatic nerve crush, mice were intraperitoneally injected with 20 mg/kg of PHA-665752 daily until sacrifice, followed by TEM and IHC. (**A**) Effects of PHA-665752 on c-met expression. Sciatic nerves were isolated at 4 d.p.i, and total proteins were prepared from three different areas followed by Western blot using specific antibodies to phosphorylated and total c-met. (n = 4 for each group). Full-length blots are presented in Supplementary Fig. S5. (**B**) Effects of PHA-665752 on HGF expression in the injured nerve. Injured nerves were isolated at 4 d.p.i and analyzed by ELISA. n = 3 for each group. (**C**) Effects of PHA-665752 on re-myelination as measured by TEM. Electron micrographs of sciatic nerves showed the cross section of sciatic nerve at 14 d.p.i, 4 mm from injury site. The white arrows indicate apoptotic cells. n = 3 for each group. Scale bar, 5 μm. (**D**) The scatter plot shows the distribution of myelin thickness versus axon diameter in PHA-995752 treated and untreated groups. The bar graphs show g-ratio value and the distribution of g-ratio. 250~300 axons, n = 3 for each group. *p < 0.05, ***p < 0.001. (**E**) Effects on axon diameter. The graph shows the distribution of axon diameter. (**F**) Effects on the number of re-myelinated axons. The number of axons was calculated from TEM photos. n = 3 for each group. ***p < 0.001. In all these experiments, values represent the mean ± S.E.M.

To study whether the increased level of HGF and c-met was involved in nerve regeneration, a specific inhibitor of c-met, PHA-665752, was used. After sciatic nerve crush, mice were intraperitoneally injected with 20 mg/kg of PHA-665752 on a daily basis until they were sacrificed. Again, the level of both total and phosphorylated c-met was increased in the distal region of sciatic nerves (Fig. 4A. lane 4). When mice were treated with PHA-665752, the level of phosphorylated c-met, but not that of total c-met, was highly reduced. The protein level of HGF was not altered by PHA-665752 treatment in the injured mouse groups, indicating that it inhibited c-met activity without affecting the expression level of HGF in the sciatic nerve (Fig. 4B).

Effects of c-met inhibition on nerve repair were tested, first by using a high-resolution transmission electron microscopy (TEM) at 14 d.p.i, which is the mid-point of the re-myelination in the nerve crush model^23^. Treatment with PHA-665752 did not produce any visible effects in sham surgery animals (Fig. 4C). In the nerve crush group injected with PHA-665752 (Crush-PHA), however, c-met inhibition significantly disrupted the nerve regeneration, as evident by the reduction in myelin thickness and myelin clearance (Fig. 4C).

To further analyze the level of myelination in the injured nerve, g-ratio was measured. After nerve crush was introduced, g-ratio value was increased to 0.8413 ± 0.00313 (Crush-DMSO), as myelin thickness was decreased. When injured mice were treated with PHA-665752, the g-ratio value was further increased to 0.8621 ± 0.003495. Furthermore, the distribution graph of g-ratio showed that Crush-PHA group was shifted right compared to the Crush-DMSO group, indicating that axons in Crush-PHA mice became hypo-myelinated compared to the Crush-DMSO group (Fig. 4D). In addition, the axon diameter distribution of the Crush-PHA group was shifted left compared to the Crush-DMSO group, indicating that axon diameters were decreased in the PHA-665752 treated mice (Fig. 4E). Furthermore, the number of re-myelinated axons in injured nerves was also decreased by PHA-665752 treatment (Fig. 4F). These data demonstrated that administration of c-met inhibitor, PHA-665752, could inhibit or delay the SCs-mediated nerve regeneration.

### Treatment of SCs with recombinant HGF protein increased the level of phosphorylated c-met and their migration and proliferation capabilities

**Figure 5:**
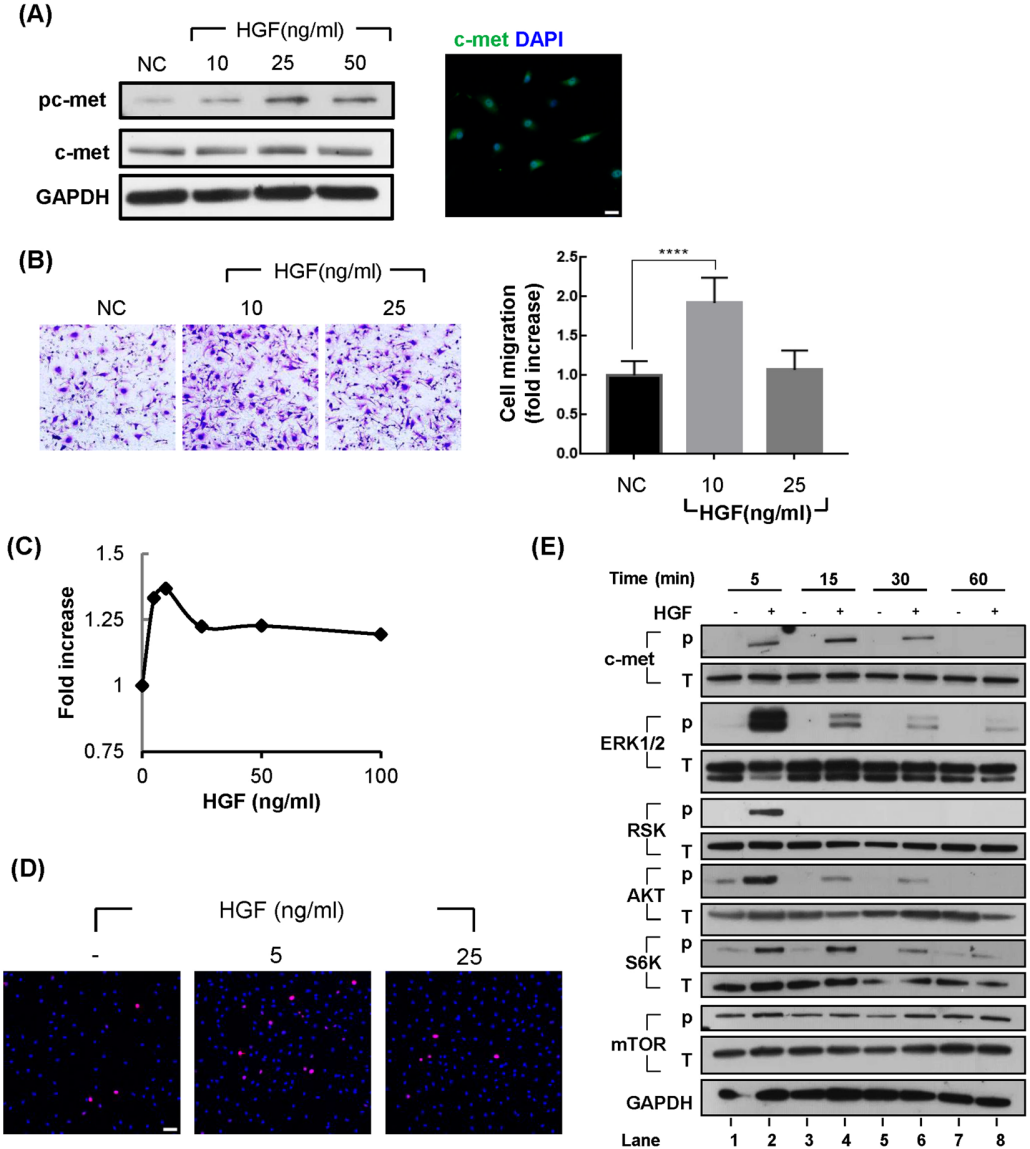
Effects of HGF on migration and proliferation of Schwann cells. Primary Schwann cells isolated from rat sciatic nerves were treated with recombinant human HGF protein to measure the effects on migration and proliferation. (**A**) Expression of c-met receptor in primary SCs. Scale bar = 25 μm. Full-length blots are presented in Supplementary Fig. S6. The samples derived from the same experiment and blots were processed in parallel. (**B**) Effects of HGF on SC migration. Primary SCs were treated with 10 ng/ml and 25 ng/ml of recombinant HGF protein, and their migration was analyzed using Boyden chambers. ****p < 0.0001. Values represent the mean ± S.E.M. of three independent experiments. (**C**) Effect of HGF on proliferation of SCs. SCs were treated with 5, 10, 25, 50, and 100 ng/ml HGF and SC proliferations were analyzed by WST-1 assay. (**D**) SCs proliferation was analyzed by immunocytochemistry assay, using an antibody to Ki67 (Red). Scale bar = 50 μm. (**E**) Signaling cascades activated by HGF treatment in SCs. Primary Schwann cells isolated from rat sciatic nerves were treated with recombinant human HGF protein. Total proteins were isolated from SCs treated with HGF at appropriate times and analyzed by Western blot using antibodies to respective proteins. Full-length blots are presented in Supplementary Fig. S7. The samples derived from the same experiment and blots were processed in parallel.

The above data suggested that HGF produced from the injury site might interact with the c-met receptor present on SCs. We tested the effect of recombinant HGF protein binding to c-met on SCs, using primary SCs isolated from an adult rat. To be certain, the expression of c-met in these cells was first tested. The basal level of the total c-met protein was readily detectable, as measured by Western blot and immunofluorescence assays, and was not changed by HGF treatment (Fig. 5A). Phosphorylated c-met was barely detectable without HGF treatment, but its level was increased when cells were treated with the HGF protein, indicating that under the normal situation, c-met is present in an un-phosphorylated form in SCs.

Because c-met expression is highly induced in distal SCs undergoing dedifferentiation, it was tested if HGF could affect migration or proliferation of SCs, as they are key features of repair SCs^24^. Primary SCs were treated with three different concentrations (10, 25, and 50 ng/ml) of recombinant human HGF protein, and its effect on phosphorylated c-met was determined by Western blot using a specific antibody (Fig. 5A). Treatment with the HGF protein highly increased the level of phosphorylated c-met. In addition, as same with the previous reports^25^, HGF also enhanced migration of SCs determined by Boyden Chamber assays (Fig. 5B). As shown in Fig. 5C, HGF also increased the proliferation of SCs, although its effects were relatively mild compared to other well-known mitogenic growth factors such as neuregulin-1 (data not shown). Induction of proliferation by HGF was also confirmed by immunocytochemistry assay, using an antibody specific for Ki67 (Fig. 5D). Taken together, these results suggested that HGF could directly interact with SCs to activate c-met and subsequently increase migration and proliferation of SCs.

### HGF selectively controls the expression of cellular genes and signaling molecules involved in nerve repair

It is well known that during dedifferentiation of SCs, there are great changes in the activity and expression level of many cellular proteins^24^. The effects of HGF in primary SCs were investigated, first on the RNA level of genes known to participate in or be affected by the nerve regeneration process. Primary SCs were treated with 25 ng/ml of HGF for 1 hour, and its effects on cellular genes were analyzed by quantitative RT-PCR and Western blot. The effects on the RNA level of those genes known to be involved in nerve regeneration are presented in Table 1. Among various transcription factors, a change in the RNA level of Egr-1 was the most prominent. The role of Egr-1 in nerve regeneration has not yet been well characterized. The RNA level of c-Fos and JunB was also increased by HGF, more than 3-fold. These molecules could form AP-1, which is widely known to control the expression of various genes taking part in SC-mediated nerve repair. HGF did not alter the expression of c-Jun and Sox10. GDNF is the most affected growth factor of the proteins tested. Among 5 inflammatory proteins, the RNA level of TNF-α and LIF, which attracts macrophages to the injury site, was increased by more than 3-fold. Overall, data from quantitative RT-PCR assays suggested that HGF could induce the expression of selective cellular genes known to play roles in the promotion of SC dedifferentiation.

**Table 1:** Effects of HGF on RNA levels of genes increased in repair SCs.

Functions		Fold increase*	STD	P-value
Transcription factors	Egr1	239.701	48.998	0.014
	c-Jun	0.872	0.068	1.000
	c-Fos	3.143	0.246	0.002
	JunB	3.827	0.244	0.001
	Sox10	1.091	0.048	0.600
	HIF1a	1.632	0.104	0.011
	Notch1	1.445	0.323	0.009
Growth factors	VEGFa	1.202	0.046	0.001
	NGF	1.055	0.191	0.724
	GDNF	4.118	0.077	0.001
Inflammatory proteins	TNFa	3.003	0.012	0.010
	LIF	7.662	0.066	0.004
	CCL2/MCP-1	1.266	0.230	0.031
	IL1b	1.682	0.099	0.001
	IL-6	2.041	0.368	0.063

Primary SCs were treated with 25 ng/ml of HGF protein for 1 hr. Total RNAs were isolated from SCs and analyzed by quantitative RT-PCR. *Fold increase after HGF treatment relative to no treatment. Values represent the mean ± S.E.M. of three independent experiments.

The effects of HGF on various signaling molecules are shown in Fig. 5D. The level of phosphorylated c-met was increased 5 min after HGF treatment, reaching the highest point at 15 min and then decreased(Fig. 5D, lanes 2, 4, 6 and 8). The level of total c-met remained unchanged throughout the experimental period. The response of phosphorylated ERK1/2 (pERK1/2) was similar to c-met, but the change was more rapid and dramatic in quantity (Fig. 5D, lanes 2 and 4). RSK, one of the downstream targets of ERK signaling was changed in a similar way to that of ERK; the level of phosphorylated RSK was highest at 5 min, and undetectable at any other time point. The level of phosphorylated AKT was also highly increased at 5 min, but to a lesser extent than that of pERK1/2. After the peak level, it was reduced slowly.

S6K is the downstream target of AKT/mTOR pathway. The phosphorylated level of S6K reached a peak at 5 min, and then was gradually reduced. The level of phosphorylated mTOR was not affected by HGF treatment, so the increase in the level of phosphorylated S6K is not due to mTOR pathway. In the same experiments, HGF did not have any effect on STAT3 and JNK, although they have been previously known to be activated by HGF in other cell types (data not shown). Taken together, these data suggested that HGF might induce the expression of selective genes involved in dedifferentiation, probably through the activation of ERK or AKT signaling.

### The ERK pathway appears to play a major role(s)

**Figure 6:**
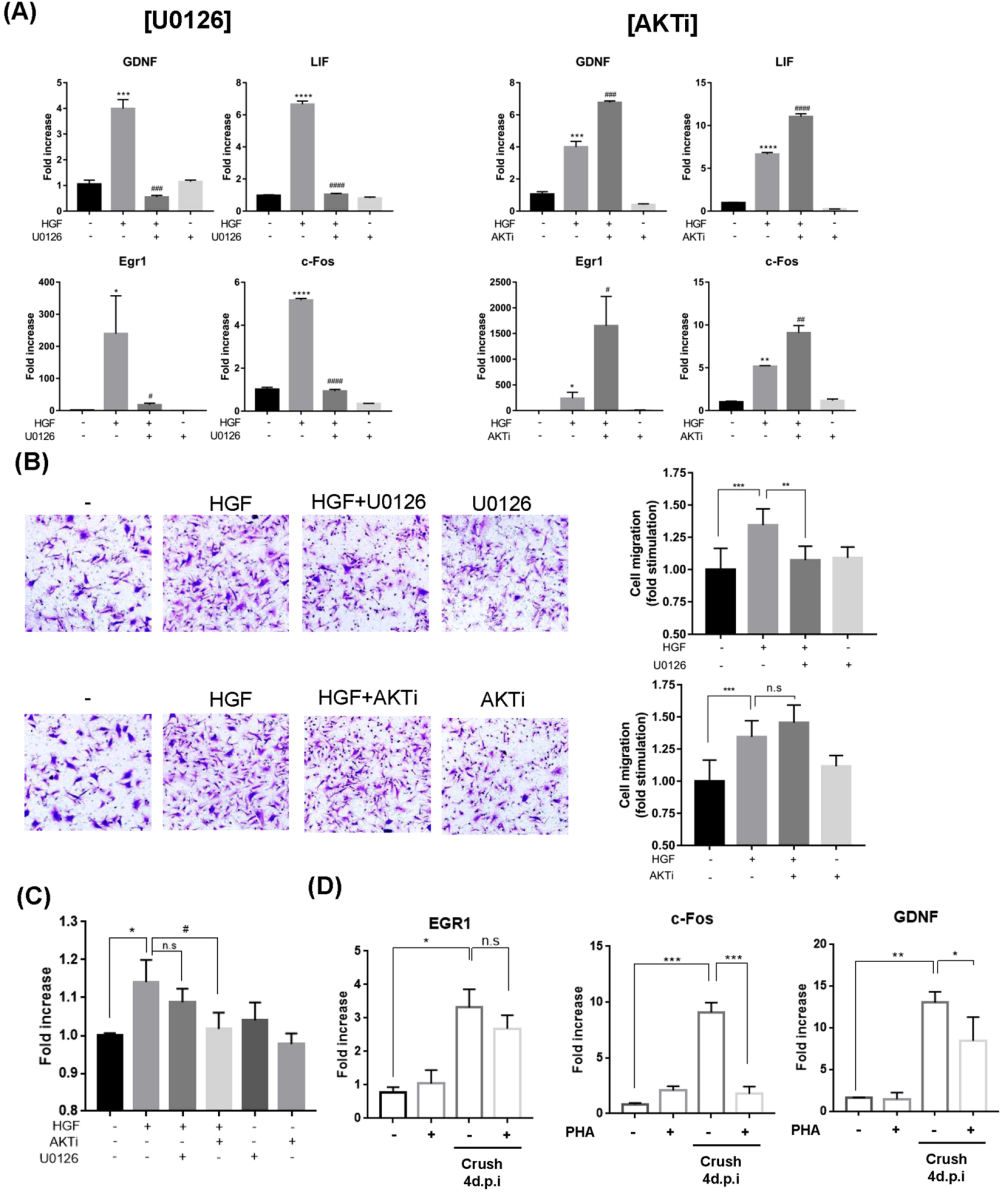
Effects of ERK and AKT inhibitors in primary SCs. Primary SCs were treated with 25 ng/ml of recombinant HGF protein in the presence of 10 μM of U0126 or 10 μM of AKTi. Total RNAs were prepared at 1 hr and subjected to quantitative RT-PCR. Effects on Cell migration and proliferation were measured by Boyden chamber and WST-1 assays, respectively. (**A**) Effects of U0126 and AKTi on the RNA level of Egr-1, c-Fos, GDNF and Lif. *p < 0.05, **p < 0.01, ***p < 0.001, ****p < 0.0001 vs. control. ^#^p < 0.05, ^##^p < 0.01, ^###^p < 0.001, ^####^p < 0.0001 vs. HGF only treated group. (**B**) Effect on SC migration. **p < 0.01, ***p < 0.001, n.s = not significant. (**C**) Effect on SC proliferation was measured by WST-1 assay. *p < 0.05 vs. control. ^#^p < 0.05 vs. HGF only treated group. (**D**) Effects of PHA-665752 on gene expression in injured nerve was measured by quantitative RT-PCR. Total RNAs were isolated from sciatic nerve at crush 4 d.p.i, *p < 0.001, **p < 0.01, ***p < 0.001. Values represent the mean ± S.E.M.

To test which of the two signaling pathways, ERK or AKT, played a more dominant role, chemical inhibitors for ERK or AKT were used. Primary SCs were treated with HGF in the presence or absence of U0126 or AKTi, an inhibitor of ERK or AKT respectively, followed by quantitative RT-PCR, Boyden chamber and WST-1 assays. As shown in Fig. 6A, an HGF-mediated increase in the RNA level of four genes (Egr1, c-Fos, GDNF, and LIF) involved in SC-mediated nerve regeneration was effectively suppressed by treatment with U0126, while the completely opposite result was obtained for AKTi, which further enhanced the expression level increased by HGF.

Similar results were obtained in cell migration assay. Treatment with U0126 also effectively inhibited HGF-induced migration of SCs, while AKTi had no effects (Fig. 6B). Interestingly, the effect on cell proliferation was different; an HGF-mediated increase in the cell number was inhibited by AKTi, but not by U1206 (Fig. 6C), indicating that AKT signaling might play a role(s) in cell proliferation rather than cell migration. These results suggested that the ERK pathway might be needed for HGF to induce migration of SCs as well as activate the expression of cellular proteins known to involve in nerve regeneration.

To prove HGF involve in regeneration process by regulating SC activation, the effect of PHA-665752 on the expression of various genes was measured in Crush model. Mice were daily injected with 20 mg/kg of PHA-665752 for four days and total RNA was isolated from sciatic nerve followed by quantitative RT-PCR. The RNA levels of c-Fos and GDNF, which were increased by HGF treatment on primary SCs, were decreased by PHA-665752 administration in injured groups (Fig. 6D). Taken together, these data suggested that HGF might have role in nerve repair by activating SCs.

### HGF overexpression induces nerve regeneration

Because the HGF/c-met pathway seemed to play a role in the nerve repair process, we tested whether exogenous administration of HGF could improve the injured nerve, as in the case of VEGF and GDNF^26–29^. We employed a gene transfer method rather than using a recombinant protein, because of the short half of the HGF protein^15^. A plasmid DNA expressing HGF, pCK-HGF-X7, was intramuscularly injected into the thigh muscles around the sciatic nerve at the time of nerve crush surgery. pCK, a plasmid DNA lacking the HGF sequence, was used as a control. pCK-HGF-X7 has previously been shown to produce two isoforms of human HGF, HGF_723_ and HGF_728_, and used in clinical studies as well as in a variety of animal models^30–33^. The expression kinetics of human HGF from pCK-HGF-X7 in mice has been well established; the protein level of HGF peaked (a ~100 ng/mg of total cellular protein scale in thigh muscles around the injection site and a ~300 pg/mg range in sciatic nerves), and then decreased to become undetectable at 14 d.p.i (data not shown).

**Figure 7:**
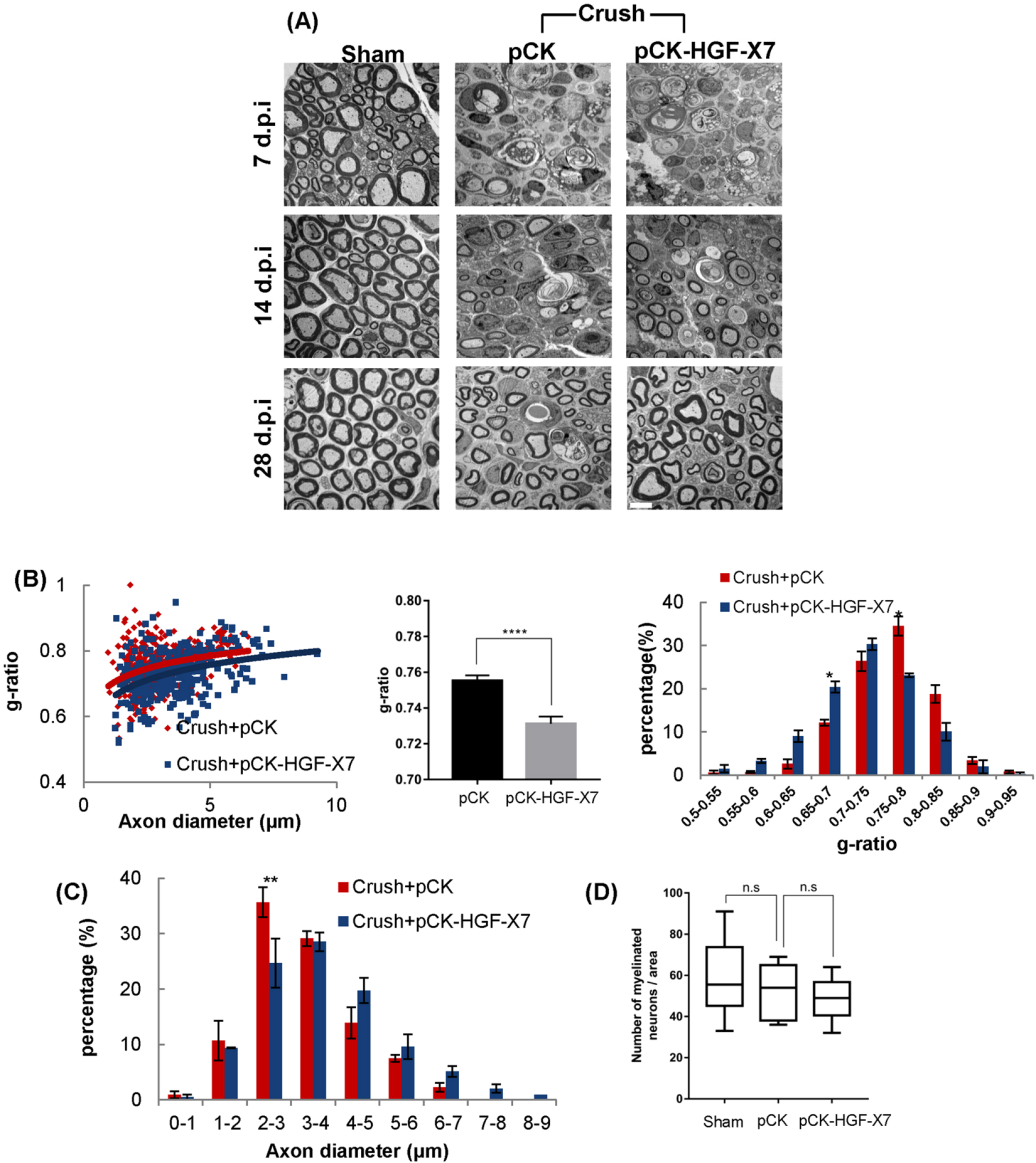
Overexpression of HGF facilitates nerve regeneration. pCK-HGFX7 was *i.m.* injected to the thigh muscle around the injured sciatic neuron at the time of nerve crush surgery. pCK lacking the HGF sequence was used as a control. (**A**) Analysis of sciatic nerves by TEM. Sciatic nerves were isolated at 7, 14, and 28 d.p.i (4 mm distal from injury site). n = 3 for each groups. Scale bar, 5 μm. (**B**) The graph shows scatter plots of g-ratio in pCK-HGFX7 and pCK control groups at 28 d.p.i. The bar graphs show g-ratio value and the distribution of g-ratio. *p < 0.05, ****p < 0.0001. (**C**) The graph presents the distribution of axon diameter. (300~400 axons, n = 3 for each group). (**D**) Effects of HGF on number of myelinated axons in injured nerves. n = 3 for each group. n.s = not significant. Values represent the mean ± S.E.M.

The effect of pCK-HGF-X7 on re-myelination was studied at 7, 14, and 28 d.p.i by analyzing the site 4 mm distal from the injury site by TEM. Little difference was found between pCK-HGF-X7 and pCK groups at 7 d.p.i, while improvements of myelin thickness and axon diameter were clearly visible at 14 and 28 d.p.i (Fig. 7A). The scatter plots of the g-ratio of each group showed that the average value of myelin thickness was higher in the pCK-HGF-X7 group compared with the pCK control group (Fig. 7B). In addition, the distribution of myelinated axon size of the pCK-HGF-X7 injected mice shifted toward the right, indicating an increase in the diameter of regenerating axons (Fig. 7C). The number of myelinated axons was not altered in both the pCK and pCK-HGF-X7 injected groups (Fig. 7D). Taken together, our data demonstrated that exogenously added HGF, delivered in the form of plasmid DNA, could improve nerve regeneration by acting on SCs.

## Discussion

In this study, we demonstrated the involvement of HGF in Schwann cell-mediated regeneration of peripheral nerves in the nerve crush model. HGF expression was highly induced in the injured and distal areas while the expression of both total and phosphorylated c-met receptor was increased almost exclusively in SCs at distal sites. When mice were treated with a c-met inhibitor, PHA-665752, re-myelination of the injured nerve was suppressed as evidenced by a decrease in myelin thickness determined by TEM analysis, indicating that HGF might have a role(s) in nerve regeneration. Treatment of primary SCs with the HGF protein enhanced migration and proliferation of SCs, and increased the expression level of neurotrophic factors such as GDNF and LIF involved in nerve regeneration after injury. As evidenced by data from the experiments involving specific chemical inhibitors, ERK or AKT signaling pathways seem to play important roles. Finally, overexpression of HGF around the nerve injury site by *i.m* injection of HGF expression plasmid DNA, pCK-HGF-X7, increased the myelin thickness and axon diameter. Taken together, our data suggest that HGF and c-met play important roles in peripheral nerve regeneration by activating SCs.

One of the most interesting observations made in this study is that c-met expression is highly induced, selectively in the distal SCs. It is well established that after nerve crush damage, most cells around the injury site undergo apoptosis, and the distal part of the nerve undergoes Wallerian degeneration. SCs present in the distal site become dedifferentiated, while a majority of those in the proximal region maintain their original differentiation status^24^. Our data indicated that only distal SCs undergoing dedifferentiation expressed a large amount of the c-met protein.

High level induction of HGF on the injury site and of the c-met protein in distal SCs appear to be two independently occurring events as treatment with a c-met inhibitor reduced the amount of phosphorylated c-met in distal SCs, while having no effect on the total amount of c-met and mouse HGF protein (Fig. 4A and B). If HGF produced from the injury site had effects on the level of total c-met expression in distal SCs, c-met expression would have been decreased by this inhibitor interacting with the surface receptor. AP-1, Pax3, and NF-kB are the major candidates responsible for the induction of c-met expression in distal SCs. These transcription factors are well known to be highly expressed in dedifferentiated SCs, and are key transcription factors driving gene expression from the c-met promoter, together with Pax7, and Ets-1^34,35^. Further studies are needed to pinpoint the main driving force(s) of c-met transcription in distal SCs.

The HGF protein was observed mainly around the injured site, but we have not yet identified the exact producer cell type(s). In naïve mice, the basal level of HGF was detected at 200 pg/mg of total protein in the sciatic nerve (Fig. 1B). Data from IHC indicated that cells present in the epineurium might be one of the major sites of HGF production after nerve injury as well as in normal, undamaged nerves. Fibroblasts, already known to produce HGF in a variety of organs, are a candidate for a major HGF producer cell type in our case, as they are present around the epineural membrane of the control nerve and are recruited to the injury site. SCs themselves do not seem to produce HGF during injury as the level of HGF expression in primary SCs was not changed when treated with a variety of stimulants including IL1β, hypoxia induced by CoCl2, and HGF itself (unpublished data). Efforts to identify the producer cell type(s) are currently underway.

Among the signaling molecules activated by HGF treatment, ERK pathway seems to play a major role in HGF-mediated nerve regeneration. The use of U0126, an ERK inhibitor, effectively suppressed HGF-mediated induction of Egr1, c-Fos, GDNF, and Lif as well as SC migration. The role(s) of AKT signaling is puzzling as treatment with AKTi increased the expression levels of those 4 genes involved in nerve regeneration, while inhibiting cell proliferation with no effect on cell migration. A bulk of literatures showed that expression of neurotrophic factors and cell migration are important in the nerve regeneration process, while SC proliferation plays no or minor role in nerve repair^5,7,36–41^. Taking several lines of data into consideration, it seems to be the ERK pathway that plays important roles in HGF-mediated nerve regeneration through SCs.

HGF overexpression around the injury site by *i.m*. injection of the HGF-expressing plasmid facilitated the re-myelination process, confirming HGF as an important player in Schwann-mediated nerve regeneration.In the case of PNS injury or disease model, there are very few cases in which re-myelination was convincingly demonstrated by transfer of neurotrophic genes. To our knowledge, there are two cases showing the effects on both axonal regeneration and re-myelination; one was the case of using NT-3 by AAV1^42,43^ and the other was the case of using GDNF by adenovirus^44^. We showed that overexpression of HGF improved myelin thickness in the injured nerve, to an extent that the g-ratio of some nerves became similar to that of the control groups. On the other hand, results from phase I and II studies, involving the HGF-expressing plasmid used in this report, in patients with diabetic peripheral neuropathy, indicated that pCK-HGF-X7 was safe and furthermore effectively reduced the pain level with a sign of recovery of the sensory function as measured by monofilament testing^30^. Taken together, HGF may be used as the platform or a starting material in developing therapeutics that can provide fundamental treatment methods for a variety of neurological diseases including peripheral neuropathy.

## Materials and Methods

### Animals

Seven-week-old male C57BL/6 mice were purchased from DaehanBioLink Inc. (Chungbuk, Korea) and used for *in vivo* analysis of protein levels and regeneration assays. Mice were housed at 23 °C with a 12-h light/dark cycle and given free access to food and water. For primary Schwann cell culture, 10-week-old male Sprague Dawley rats were purchased from DaehanBioLink Inc. (Chungbuk, Korea) and were euthanized by CO_2_ gas on the day of experimentations. All experimental procedures were performed in compliance with the guidelines set by the University Animal Care and Use Committee at Seoul National University with special attention given to minimizing animal pain

### Nerve crush

Nerve crush was performed as previously described^45^. For nerve crush, C57BL/6 mice were anesthetized with isoflurane. All surgical protocols were approved by the Institutional Animal Care and Use Committee. For each mouse, the area above the right thigh was shaved and sterilized with Povidone and 70% EtOH. The right sciatic nerve was exposed by a small incision made to the skin and muscle. Two millimeter of the nerve was crushed for 15 seconds with fine hemostatic forceps (FST), which had been dipped in powdered carbon (Sigma) before use to mark the crush site^45^. Then the incision was sutured using a 5–0 black silk suture (ALEE). PHA665752, a c-met inhibitor, was intraperitoneally injected into each mouse in a dosage of 20 mg/kg/day. Two hundred micrograms of pCK or pCK-HGF-X7 were diluted in 100 μl PBS and directly injected once to the thigh muscles around the sciatic nerves.

### Sciatic nerve preparation

After mice were sacrificed at appropriate time points, ipsilateral sciatic nerves were isolated. Injured sciatic nerve was divided into three areas; proximal (2 mm above the injury site), injury (the site crushed by hemostatic forceps and labeled with powdered carbon) and distal (2 mm below the injury site) sites.

### Antibodies

The following antibodies were used: anti-S100b (1:500, ref Z0311, Dako), anti-GFAP (1:500, ref M0761, Dako), anti-phosphocMet (Y1234/1235) (1:250, ref 3077, Cell Signaling), anti-phospho ERK1/2 (1:1000, ref 9106, Cell Signaling), anti-ERK(1:1000, ref 4695, Cell Signaling), anti-phospho S6K (1:500, ref 9205, Cell Signaling), anti-S6K (1:1000, ref 2708, Cell Signaling), anti-phospho AKT (1:1000, ref 13038, Cell Signaling), anti-AKT (1:1000, ref 9272, Cell Signaling), anti-phosphomTOR(1:1000, ref 2971, Cell Signaling), and anti-mTOR(1:1000, ref 2983, Cell Signaling), anti-HGF (1:500, ref MA5-14160, Thermo scientific), anti-GAPDH (1:5000, ref ab9485, Abcam), anti-cMet (1:1000, ref SAB4300599, Sigma), anti-Rabbit IgG secondary antibody Alexa Flour 555 (1:500, ref A-31572, Thermo Scientific), anti-Rabbit IgG secondary antibody Alexa Flour 488 (1:500, ref A-21206, Thermo Scientific), anti-mouse IgG secondary antibody Alexa Flour 555 (1:500, ref A-31570, Thermo Scientific), anti-mouse IgG secondary antibody Alexa Flour 488 (1:500, ref A-11006, Thermo Scientific), anti-mouse IgG HRP (1:10000, ref A0168, Sigma), and anti-rabbit IgG HRP (1:10000, ref RABHRP1, Sigma).

### Transmission Electron Microscopy (TEM)

The animals were anesthetized with isoflurane. Mice were fixed by cardiovascular perfusion with 2% paraformaldehyde +2% glutaraldehyde in 0.1 M PBS. After fixation, the right ipsilateral sciatic nerve was prepared, and the tissue was immediately incubated in fixation solution for 4 hrs at 4 °C, followed by treatment with 1% osmium tetroxide in 0.1 M PBS for 2 hrs at 4 °C for post-fixation. Fixed tissues were washed twice in distilled water, and en bloc staining was performed with 2% aqueous uranyl acetate (UA) overnight at 4 °C in the dark. On the subsequent day, tissues were dehydrated by a serial passage in 30%, 50%, 70%, 80%, 90%, 100%, 100%, and 100% ethanol for 10 min each. Before resin embedding, the dehydrated tissues were suspended in propylene oxide twice for 10 min at 4 °C and incubated in a series of propylene oxide plus Spurr resin mixtures (2:1, 1:1, 1:2) for 1 hr each. Tissues were then embedded with 100% resin overnight. Samples were polymerized with Spurr resin embedded in molds and incubated in a 70 °C dry oven overnight. Sample sectioning and visualizing images by Transmission Electron Microscopy were done by NICEM (Seoul National University, Korea). The lengths of axon diameter and myelin thickness, and the number of regenerating axons were manually determined by Image J program (NIH).

### Immunohistochemistry (IHC)

Immunohistochemical analyses were performed as previously described^46^. Briefly, mice were fixed in 4% paraformaldehyde and cryo-sectioned to 12 μm thickness. Sections were washed in 0.1 M PBS (pH7.4) twice, then blocked for 1 h with PBS containing 5% fetal bovine serum (Corning), 5% donkey serum (Jackson ImmunoResearch Laboratories), 2% BSA (Sigma) and 0.1% Triton X-100. Samples were incubated with primary antibodies diluted in blocking buffer overnight at 4 °C. Sections were washed four times in PBS and incubated for 1 hr at room temperature with secondary antibodies (Invitrogen) diluted in PBS. Immunostained samples were further washed 6 times and counterstained with Hoechst 33342 (Sigma) for nuclear staining. The fluorescence images were obtained using a Zeiss LSM 700 confocal microscope (Zeiss, Oberkochen, Germany).

### Schwann cell culture

Schwann cell cultures were performed as previously described in Kaewkhaw *et al*.^47^. After isolation, primary Schwann cells were expanded in the culture medium [DMEM–d-valine (Welgene, Korea) +2 mM glutamine (Invitrogen) +10% FBS (Corning) +1% N2 Supplement (Invitrogen) +20 µg/ml bovine pituitary extract (Sigma) +5 µM forskolin (Sigma) +100 U/ml penicillin/100 µg/ml streptomycin (Invitrogen) +0.25 µg/ml amphotericin B (Sigma)]. Primary SCs were used only until 3~4 passage. To study the HGF signaling pathway, primary Schwann cells were incubated in the DMEM medium containing 1% FBS + PSG for 4 hrs for serum starvation. All reagents, including inhibitors and recombinant human HGF (R&D) were diluted in 1% FBS + PSG DMEM media prior to treatment.

### Immunofluorescence assays

Immunofluorescence assays were performed as previously described^46^. Briefly, primary adult SCs were fixed using a 4% paraformaldehyde solution for 15 mins and washed by PBS three times. These were then permeabilized with 0.5% Triton X-100 in 0.1 M PBS (pH7.4) for 15 minutes. The samples were washed with PBS three times and, followed by blocking with [10% FBS + 2.5% donkey serum in PBS] for 1 hour. The samples were then incubated overnight at 4 °C with primary antibodies diluted in the blocking solution. After 3 washes with PBS, the samples were incubated with fluorescence dye conjugated secondary antibodies for 1 hour at room temperature. Nuclear staining was done using 1 ug/mL Hoeschst 33248 (Sigma).

### Protein preparation

For Western blot analysis, sciatic nerves and primary Schwann cells were prepared using RIPA buffer containing a protease inhibitor (Roche), phosphatase inhibitor cocktail (Roche) and 1 mM PMSF. Equal amounts of protein samples were subjected to SDS-PAGE on 10% polyacrylamide gels, and transferred to polyvinylidene fluoride membranes (Millipore, Bedford, MA, USA). The membranes were blocked with 0.1% TBST containing 1% (w/v) BSA (Invitrogen, Carlsbad, CA), and incubated with primary antibodies diluted in a 3% BSA blocking solution overnight at 4 °C. Membranes were then incubated with HRP-conjugated anti-mouse or anti-rabbit IgG (1:100,000; Sigma) for 1 hr, and protein bands were visualized with ECL (Millipore, Billerica, MA, USA) and X-Omat film (Kodak, Rochester, NY).

### ELISA

Mouse HGF, and mouse VEGF ELISA kits were purchased from R&D Systems (R&D Inc.). All ELISAs were performed according to protocols provided by R&D Inc. When *in vivo* samples were prepared, sciatic nerves were isolated and homogenized in RIPA lysis buffer containing a protease inhibitor (Roche), phosphatase inhibitor cocktail (Roche) and PMSF (Sigma) by using polypropylene pestles (Bel-Art Scienceware). After preparation, samples were centrifuged at 12,000 rpm for 10 min at 4 °C. The supernatants were used to detect mouse HGF.

### RNA preparation and Quantitative RT-PCR

Total RNAs were prepared from sciatic nerves and cultured cells using Trizol reagent (Invitrogen), and cDNAs were synthesized from 1 mg of respective RNA samples by using an oligo (dT) primer and AMV-RT enzyme (Roche, Indianapolis, IN). Real-time quantitative RT-PCR was performed with SYBR green (Takara Bio), using the Smart Cycler System’s (Takara) with the following protocol: 30 s at 95 °C, followed by 40 cycles of 5 s at 95 °C and 30 s at 60 °C. The sequences of synthesized PCR primer sets (Bioneer Co. Ltd., Seoul, Korea) for rat 18 s were 5′-CGGCTACCACATCCAAGGAA-3′ and 5′-GCTGGAATTACCGCGGCT-3′; for rat Egr1 were 5′-AGAAGGCGATGGGTGGAGACGA-3′ and 5′-TGCGGATGTGGGTGGTAAGGT-3′; for rat c-Jun were 5′-TGAAAGCGCAAAACTCCGA-3′ and 5′-TGTGCCACCTGTTCCCTGA-3′; for rat c-Fos were 5′-TCCCAGAGGAGATGTCTGTG-3′ and 5′-GGCTCCAGCTCTGTGACCAT-3′; for rat JunB were 5′-CCGGATGTGCACGAAAATGGAACAG-3′ and 5′-ACCGTCCGCAAAGCCCTCCTG-3′; for rat Sox10 were 5′-GCTATCCAGGCTCACTACAAG-3′ and 5′-ACTGCAGCTCTGTCTTTGG-3′; for rat Notch1 were 5′-GCACCTGCATTGATGATGTC-3′ and 5′-CTCCTTGCATACCCCACTGT-3′; for rat VEGF-a were 5′-GAGTTAAACGAACGTACTTGCAGA-3′ and 5′-TCTAGTTCCCGAAACCCTGA-3′; for rat NGF were 5′-TCAACAGGACTCACAGGAGCA-3′ and 5′-GGTCTTATCTCCAACCCACACAC-3′; for ratGDNF were 5′-CTGACCAGTGACTCCAATATGC-3′ and 5′-GCCTCTGCGACCTTTCCC-3′; for rat TNF-α 5′-CCAGGAGAAAGTCAGCCTCC-3′ and 5′-TCATCACAGGGCTTGAGCTC-3′; for fat LIF 5′-TCAACTGGCTCAACTCAACG-3′ and 5′-AAAGGTGGGAAATCCGTCAT-3′; for rat MCP-1 5′-GCAGGTCTCTGTCACGCTTCT-3′; for rat MCP-1 5′-GGCTGAGACAGCACGTGGAT-3′; for rat IL1b 5′-ATATGTTCTCAGGAGATCTTGGAA-3′ and 5′-TGCATCATCGCGTTCATACAA-3′; for rat IL-6 5′-AGGAAGGCAGTGTCACTCATTGT-3′ and 5′-CTTGGGTCCTCATCCTGGAA-3′; for mouse GAPDH were5′-CTGGAAAGCTGTGGCGTGATR-3′ and 5′-CCAGGCGGCACGTCAGATCC-3′; for mouse GDNF 5′-TCTCGAGCAGGTTCGAATGG-3′ and 5′-AAGAACCGTCGCAAACTTTA-3′; for mouse LIF 5′-ACGCGTGTCAGCGACAAA and 5′-GGCAGCCAGTCCTGAGATGA-3′.

### Migration assay

Primary Schwann cells (2.5 × 10^4^ cells/well) in 1% FBS media were seeded on Costar filter chambers (Bottom diameter: 6.5 mm, 8 μm pore size, Costar, Cambridge, MA) which had been coated with poly-L-lysine and laminin. Cells were allowed to migrate for 3 hr 30 mins. Migrated cells were fixed using a 4% paraformaldehyde solution for 15 mins and then stained with crystal Violet solution (0.2% crystal violet in 20% Methanol) for 30 mins. The number of cells in each image was counted by using Image-J cell counting program.

### WST-1 assay

WST-1 assay was performed according to protocols provided by CellVia cell viability assay kit (Abfrontier). Primary SCs were seeded in 96 well (0.5 × 10^4^ cells/well) and cultured in presence or absence of HGF for 24 hours.

### Statistical analysis

All statistical analyses were performed using GraphPad Prism software version 7.0. Statistical significance was estimated by one-way ANOVA with Tukey correction or Student’s t-test. Sample sizes are as described in the Fig. legends. Data were considered statistically significant if the p-value is <0.05.

### Data availability

All data generated during this study are included in this published article (and its Supplementary Information files).

## Electronic supplementary material

Supplementary information
