# Supplementary material for: Hepatocyte Growth Factor (HGF) Promotes Peripheral Nerve Regeneration by Activating Repair Schwann Cells
Source: Sci Rep. 2018 May 29;8:8316. doi: 10.1038/s41598-018-26704-x (PMC5973939; doi:10.1038/s41598-018-26704-x)
Supplement: Supplementary file 1 — Supplementary information [file 41598_2018_26704_MOESM1_ESM.pdf]

Hepatocyte Growth Factor (HGF) Promotes Peripheral Nerve Regeneration by Activating Repair Schwann Cells

Kyeong Ryang Ko[1,2], Junghun Lee[2], Deokho Lee[2], Boram Nho[1] and Sunyoung Kim[1,2]*

[1]School of Biological Sciences, Seoul National University, Seoul 08826, Korea,

[2]ViroMed, Co., Ltd, Seoul 08826, Korea

* Correspondence should be addressed to Sunyoung Kim (sunyoung@snu.ac.kr)

Corresponding author's

Address: 504-208, School of Biological Sciences, Seoul National University, Seoul 08826, Korea

Telephone: +82-2-880-7529

Fax: +82-2-875-0907

Email: sunyoung@snu.ac.kr

Short title: The role of HGF in peripheral nerve regeneration

Supplementary Figure S1

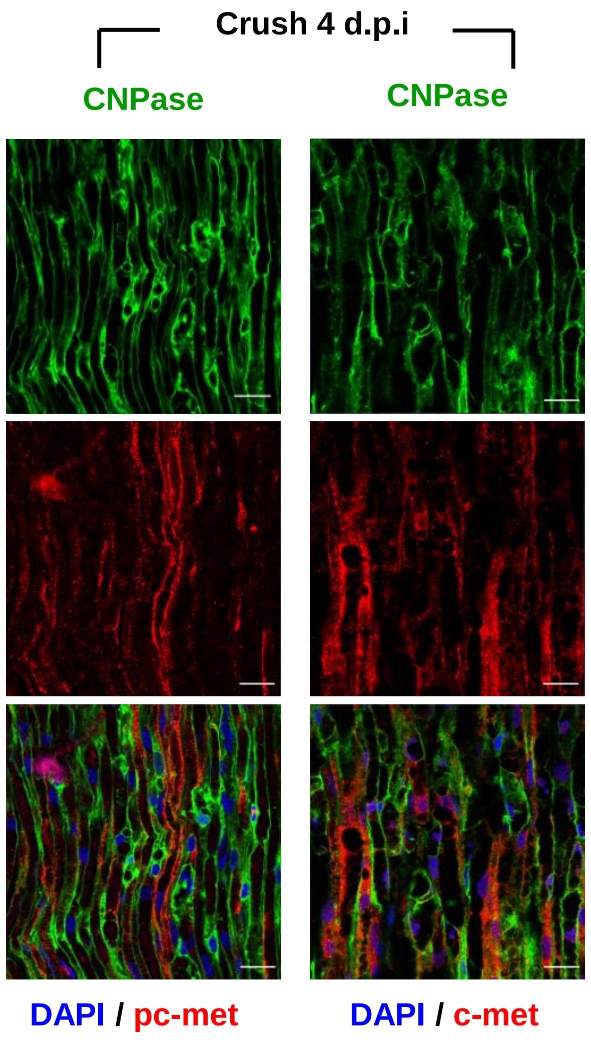

Supplementary Figure S1. Identification of cell types expressing c-met. The distal sites of injured sciatic nerves were analyzed by IHC using antibodies to CNPase (green), phosphorylated c-met and total c-met (red). Injured sciatic nerves were prepared at crush 4 d.p.i. Nuclei were counterstained with Hoechst (blue). n = 3 for each group. Scale bar = 20μm

Supplementary Figure S2

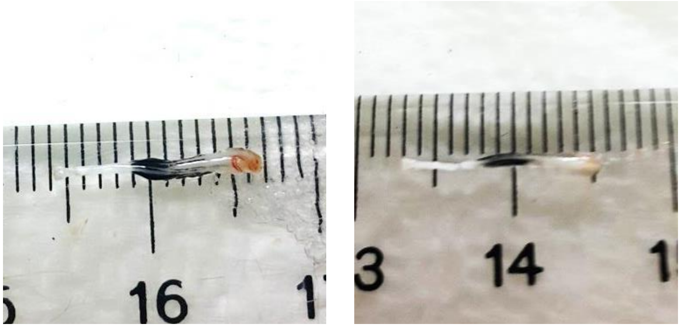

Supplementary Figure S2. Identification of the injury site of sciatic nerve. The injury sites of sciatic nerves were marked by powdered carbon.

Supplementary Figure S3

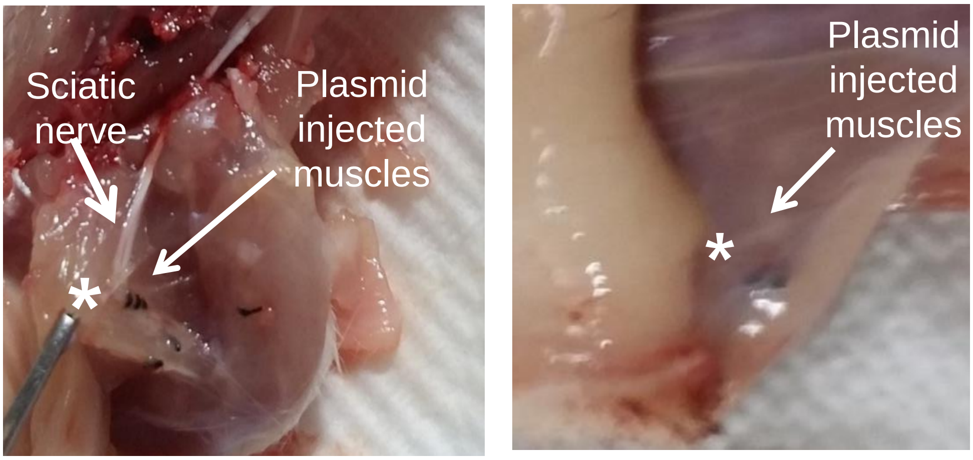

Supplementary Figure S3. Injection site of plasmid DNA. After nerve crush model was introduced, 200μg of plasmid DNA diluted in 100μl of PBS was injected to thigh muscles around the sciatic nerves. White Asterisk (*) indicated the injection site.

Supplementary Figure S4

(A)

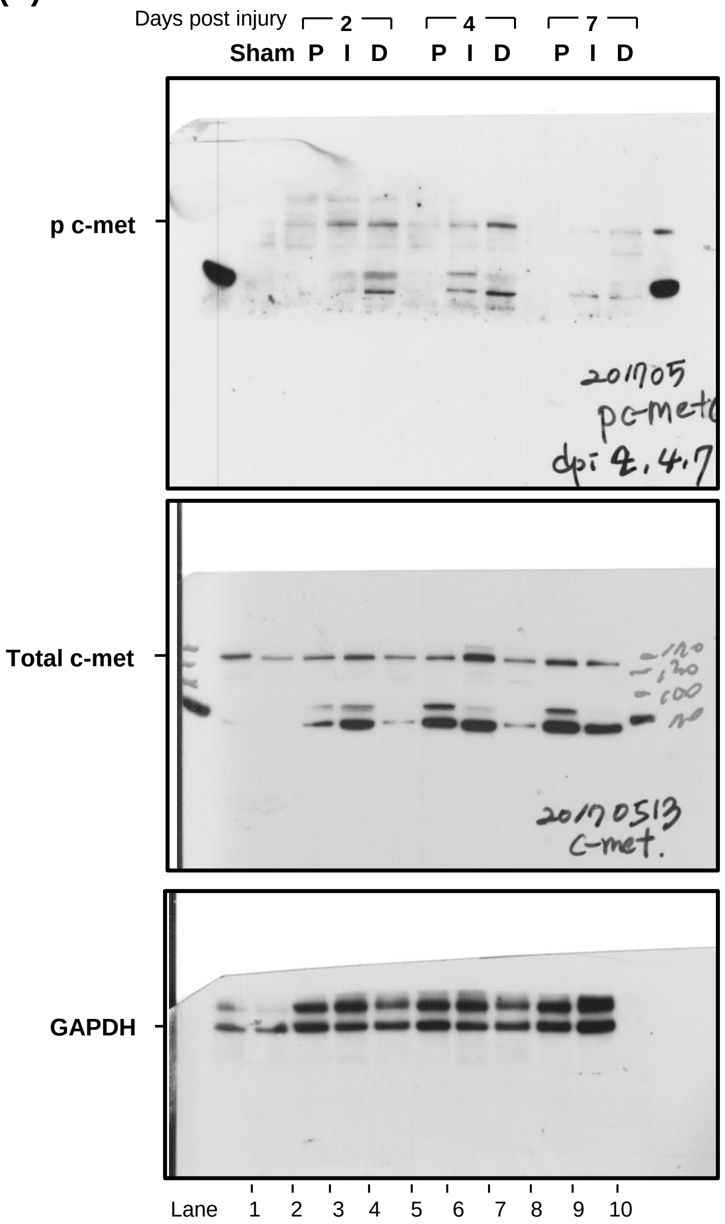

Supplementary Figure S4

(B)

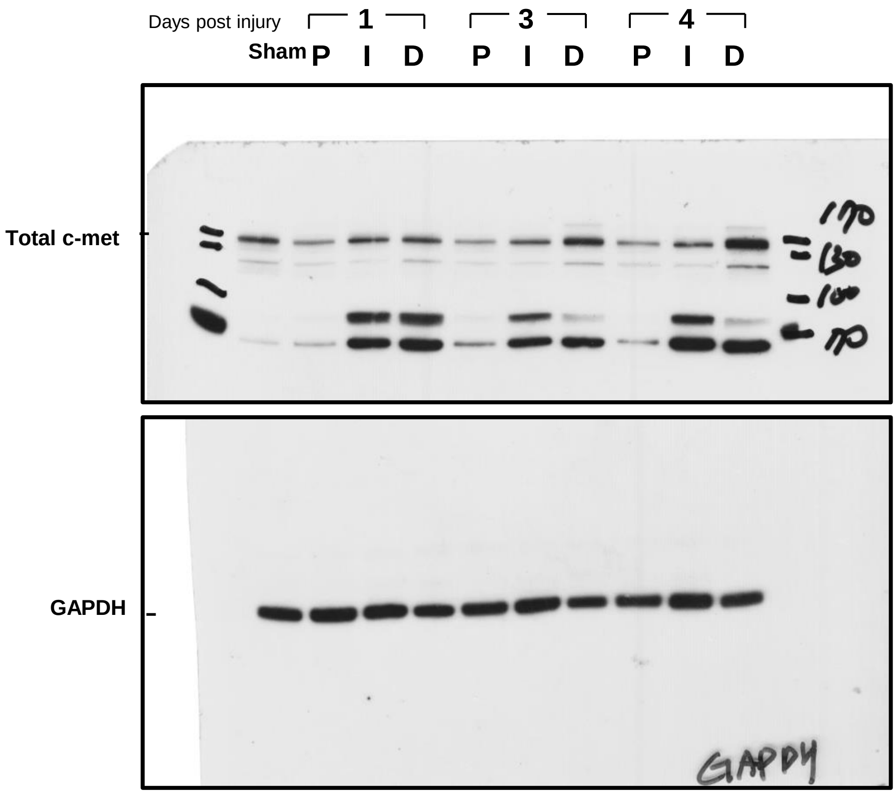

Supplementary Figure S4. Full representative Western blots. (A) Full Western blot for Figure 2A and 2B. (B) Full Western blot for Figure 2B.

Supplementary Figure S5

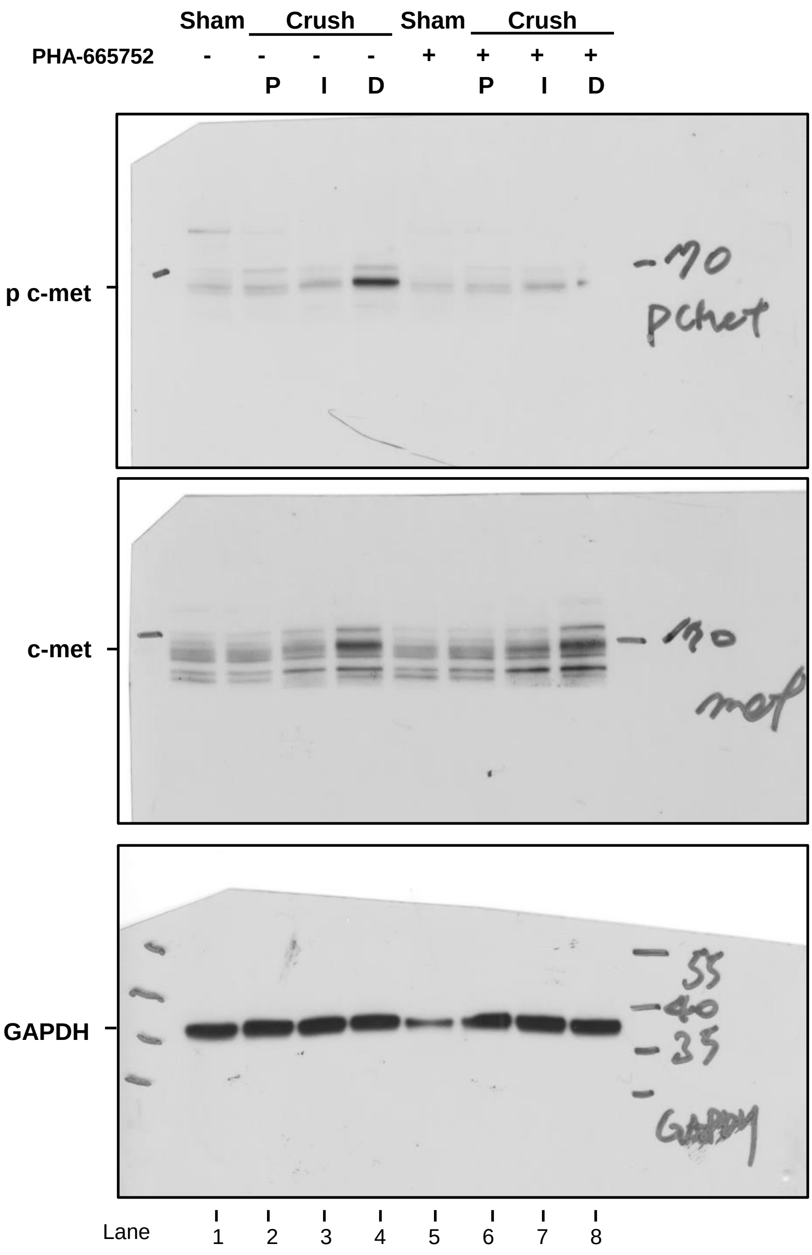

Supplementary Figure S5. Full representative Western blots. Full Western blot for Figure 4A.

Supplementary Figure S6

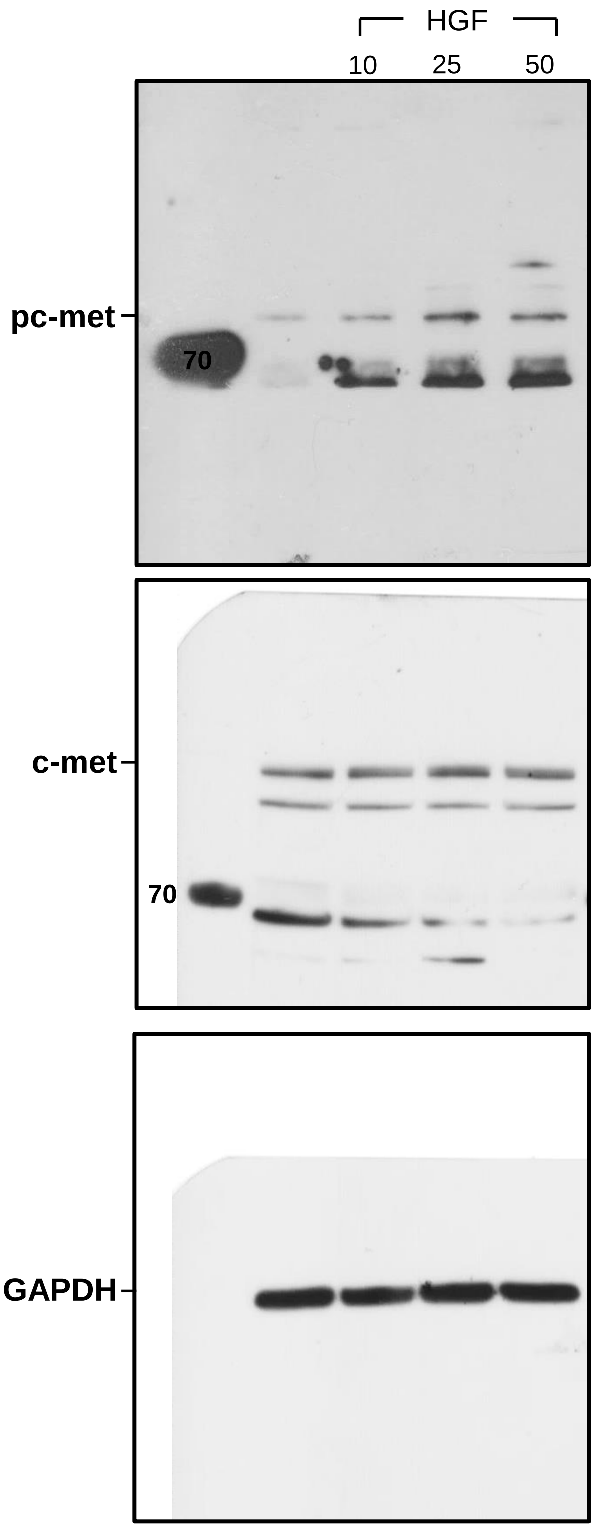

Supplementary Figure S6. Full representative Western blots. Full Western blot for Figure 5A.

Supplementary Figure S7

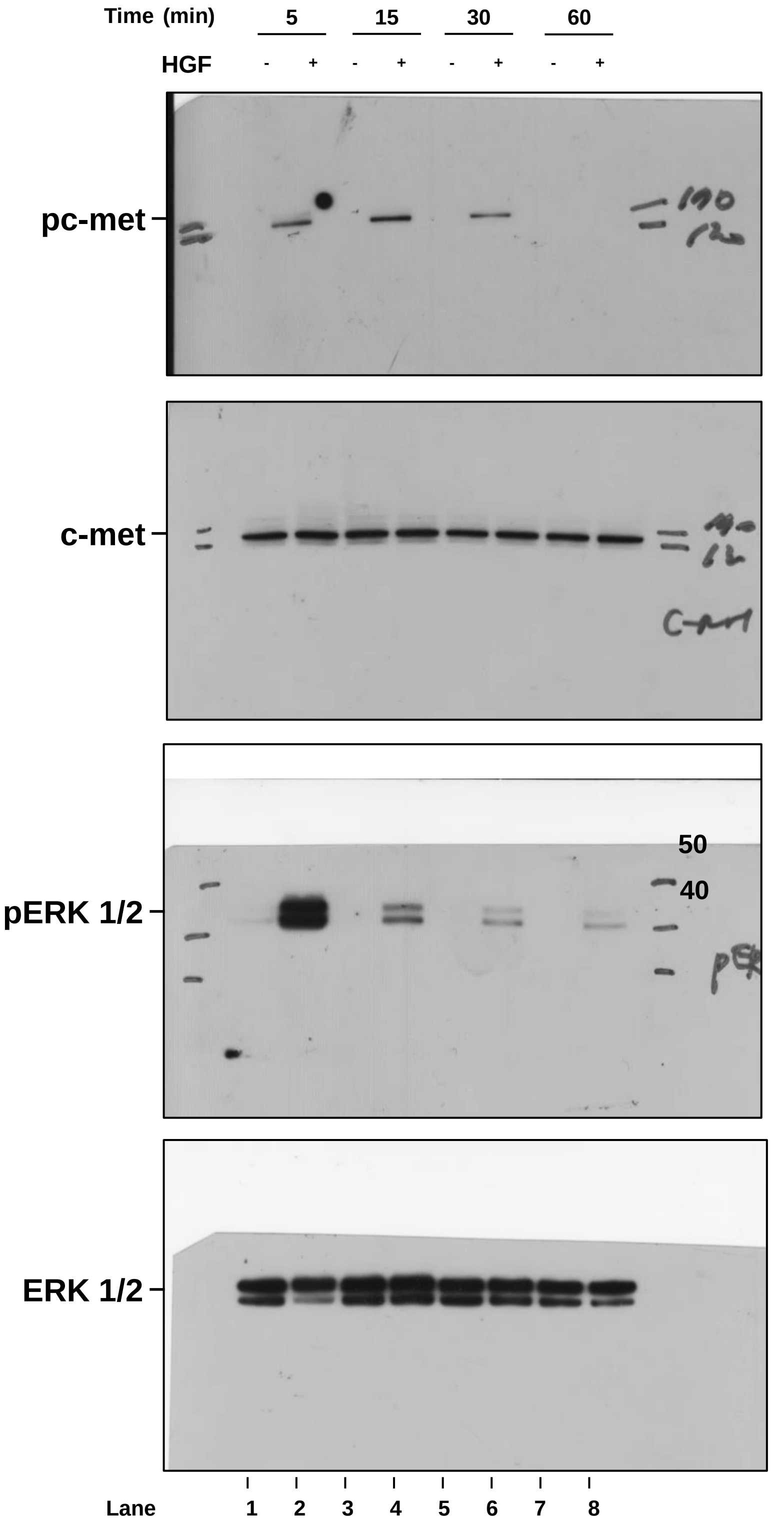

Supplementary Figure S7

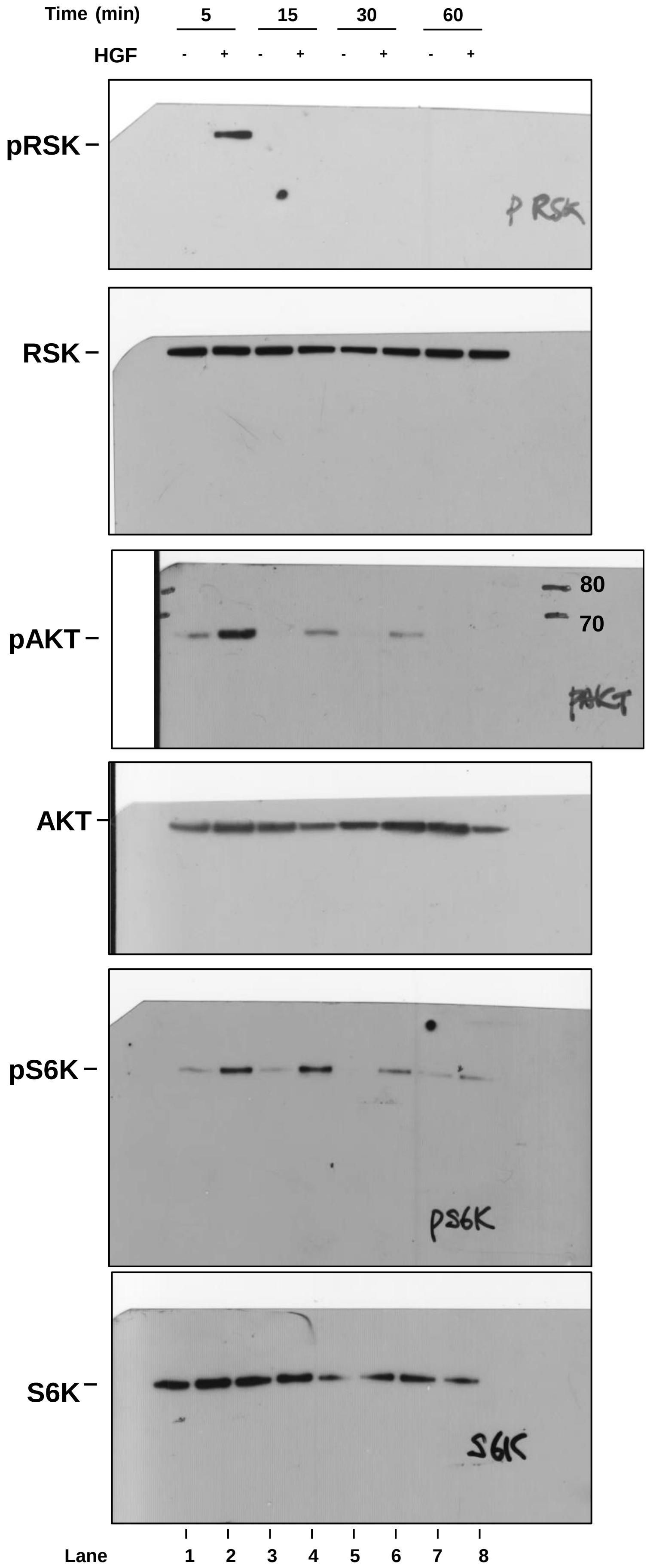

Supplementary Figure S7

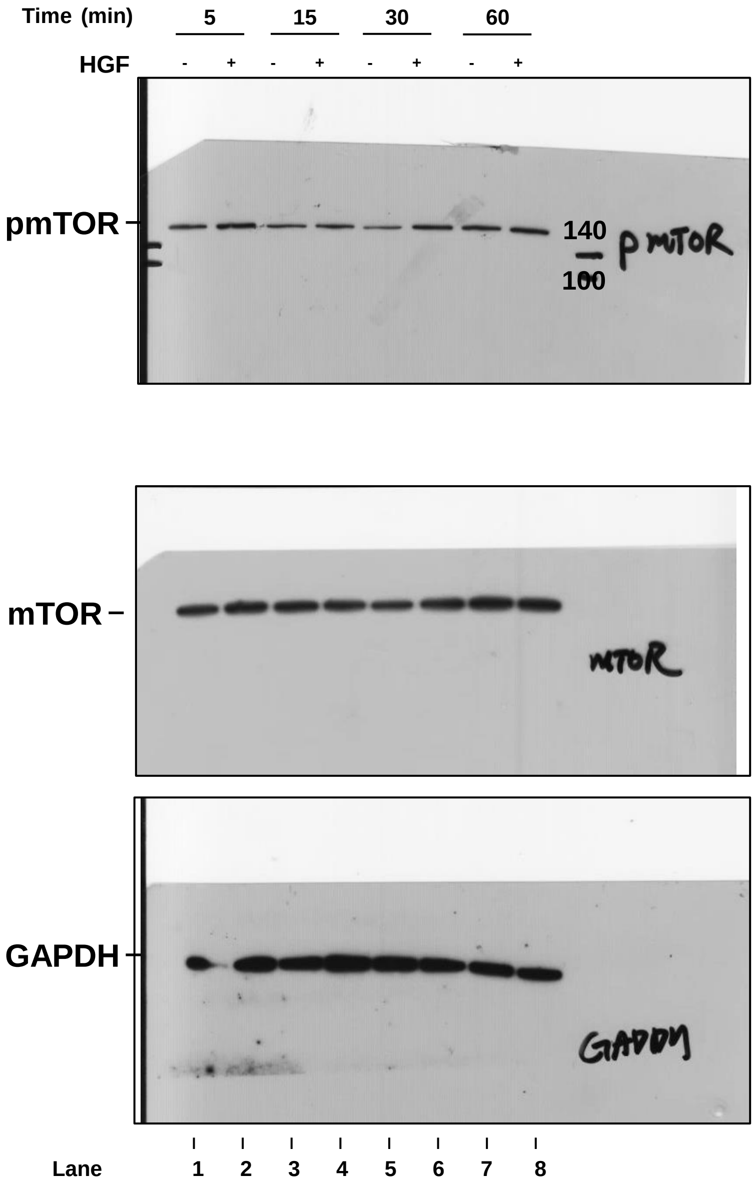

Supplementary Figure S7. Full representative Western blots. Full Western blot for Figure 5E.
